# The Week's Work

**Published:** 1910-02-05

**Authors:** 


### The Week's Work.

### THE BRADFORD OUTLOOK.

For nearly twenty years the need for a new general hospital at Bradford has been commented upon and recognised. For various reasons the matter has hung fire, but at last, thanks to the energy and public spirit of the late Lord Mayor, Mr. James Hill, and to that of the present Lord Mayor, who is showing commendable energy and interest, there is every prospect that the building of the new infirmary will speedily be proceeded with on modern and up-to-date lines. The position at Bradford is unique from the circumstance that the excellent work done by its two special hospitals is hampered by inadequate buildings. The position presented is one, therefore, which gives the citizens of Bradford an opportunity of making the whole of their hospitals the most modern and up-to-date institutions to be met with, at any rate in the North of England. It is a good sign that all classes are interested in the work. The working classes, who have a special hospital fund of their own, which raises between £7,000 and £8,000 a year for the Bradford hospitals, are interesting themselves warmly in the Building Fund of the new infirmary, to which they are contributing handsomely. During the week, for example, the Cleckheaton Co-operative Society has contributed £250, and the Brighouse Co-operative Society fifty guineas to this fund. The best available site has been privately purchased by a member or members of the Hospital Committee, and we hope that every citizen, whatever his or her station in life, may be moved to lend a hand to the great work of providing Bradford with a system of hospitals which should, when completed, present many features so novel and excellent that they will constitute types full of interest for many years to come for the study of all who have new hospitals to provide.

### WAKE UP! ABERDEEN.

The Aberdeen Trades Council at its meeting on the 28th ult. brought out a most important point in connection with the extension of the City Hospital. It appears that, like many other towns, the men attached to several trades, and especially painters, have to endure the miseries attendant upon unemployment at this season of the year. The Trades Council urged upon the City Council, which is responsible for the building of the City Hospital, the importance of giving employment during the present slack time to artisans in Aberdeen, by pushing forward the building operations at the City Hospital. It appears, however, that the City Council is not favourable to this view, for in a communication from the Town Clerk it is stated that the floors of the extensions were to be laid with pitch-pine, and it was not advisable to lay this description of flooring during the winter season. The President of the Trades Council very properly stated that the Town Clerk's letter was not satisfactory, and one member pointedly stated it was bluff to say pitch-pine floors could not be laid in the winter season. We entirely agree with this speaker, Mr. Daniel Edmonstone, for the season of the year has little or nothing to do with the laying down of flooring in a hospital building which has been properly prepared and dried for the purpose. The fact that pitch-pine is being used, though pitch-pine is a most undesirable material for hospital floors, cannot be accepted as a valid reason for delaying the work. Pitch-pine has proved unsatisfactory where it has been used for floors in this sense, and American willow is a cheaper and better wood for the purpose. Be this as it may, we hope that the members of the Aberdeen Corporation will promptly call the Town Clerk to order, that the Trades Council will, if necessary, call a public meeting, and that every effort will be made to cause the extensions at the City Hospital to be proceeded with without a moment's delay in the best interests of the artisans of Aberdeen and all the other citizens who are dependent upon the City Hospital for the accommodation they may require in the day of ill-health or incapacity.

### A PUBLIC-SPIRITED MAYOR.

When Sir Henry Burdett inspected the Chester Infirmary he made a brief entry in the visitors' book calling attention to the fact that the hospital building was over 150 years old, and without the possibility of providing the essentials which a modern hospital demands. He asked for one man in the city and county of Chester with the spirit and energy to see that this ancient city was provided with a new and up-to-date hospital worthy of its best traditions. The Mayor of Chester, Mr. Alderman D. L. Hewitt, took the chair at the annual meeting of governors on the 25th ult., when Sir Henry's remarks came up for discussion, as they were reproduced in the report of the Board of Management submitted to the meeting. Colonel Evans Lloyd, the Chairman of the Committee, alluding to this entry, asked for a millionaire to come forward and provide not less than £60,000, which he estimated as the cost of rebuilding the Infirmary. He stated, too, that to scrap all the existing buildings was rather a large order, considering that the Committee had spent from £20,000 to £30,000 during the last 20 years on alterations and improvements. The matter would probably have rested there had it not been for the Mayor, who, in speaking to a vote of thanks, declared that the citizens must be prepared for a drastic report from Sir Henry Burdett, and that he was informed by members of the medical profession that many of Sir Henry's contentions were unfortunately true. The report referred to by the Mayor has not yet been published; but we may here point out that, if the Mayor and Corporation co-operate with the governors, the present Infirmary may be modernised and brought up to date on a plan identical with that pursued at Northampton for an expenditure which need not probably exceed £25,000.

### THE NORTH STAFFORDSHIRE INFIRMARY.

With reference to our remarks, published in our issue of January 15, p. 459, we are glad to be in a position to state that the members of the Reconstruction Committee, of which Mr. W. D. Spanton, F.R.C.S., is the chairman, have shown commendable energy and devotion in the discharge of the important duties which have been entrusted to them by the governors. They have made a careful and detailed inspection of every part of the existing buildings and of the site, and they are evidently determined to bring up a report and proposals which will make this hospital worthy of the best traditions of our voluntary hospitals. They have already obtained the sanction of the Committee to the reconstruction and modernising of the laundry and heating arrangements of the entire hospital; they have asked for the cooperation of Sir Henry Burdett, and are at present engaged upon the preparation of an exhaustive scheme for the complete reconstruction of those portions of the institution which urgently call for such treatment. The Committee on By-laws has, unfortunately, lost the services of its chairman, a most excellent and valued colleague; but we hope his place may be taken by some other representative and able man, and that within the next month the Committee may have completed its labours and brought up regulations which will provide for an increase in the medical staff upon an adequate basis, and secure the more thorough and economical administration of the whole establishment for the future. If the Committee obtained a copy of the regulations and by-laws of the General Hospital, Birmingham, their work might be greatly simplified and aided.

### HOSPITAL FINANCE IN WALES.

We publish in another column a further letter from the General Superintendent of the Cardiff Infirmary. It appears from Mr. Leonard D. Rea's explanation that the overdraft is largely a matter of account. We gather that the bankers who have advanced the £24,000 on current account have in their hands money belonging to the infirmary to the credit of the new wing and extensions account, which exceeds this overdraft. We presume, therefore, that the annual expenditure is not increased materially, if at all, by bank charges and interest, in which case the treasurer and committee seem to have done themselves less than justice by their reticence at the annual meeting. In any case, we hope that the steps now being taken to increase the income of the Cardiff Infirmary may prove so successful that in future years there may be no possibility that an overdraft at the bankers will be required.

### OVERCROWDED WARDS.

In old days it used to be a common complaint that the honorary medical staff, or some of them, of most hospitals had a habit of insisting upon the admission of cases to particular wards, when all the beds assigned to those wards were already occupied. This led to the introduction of more beds and cots, and sometimes, when the system was in vogue of certain fixed days in each week as taking-in days, beds were made up on the floor and even had to be placed in the corridors. All this gave rise to very grave abuses, not the least of which was the overworking of the nurses and other attendants upon the sick, to say nothing of the faulty hygienic conditions which necessarily prevailed. As hospital committees have been strengthened by the selection of laymen of influence and importance, who take an active personal interest in the efficiency of the hospital with which they associate themselves, the committees have very properly taken steps to prevent the abuses referred to, and have forbidden the overcrowding in the wards. Particulars as to the number of patients, the cubic and superficial space per bed, and the number of beds allotted to each ward are painted upon the ward doors or printed upon cards hung up in a conspicuous position in each ward. There are, however, unfortunately, still hospitals where the committees do not appear to exercise this active control, as they should do, over the administration of some of the voluntary hospitals. We found, for instance, at the German Hospital, Dalston, the other day that all the wards we entered were overcrowded and the atmosphere of some of them was bad. The children's ward especially was overcrowded, and in most of the wards there appeared to be a disregard of the importance of superficial space, so that the beds were crowded together, and the hygienic and atmospheric conditions of parts of the hospital left much to desire. We hope the committee of the German Hospital will have this matter under its consideration at an early date and that steps will be taken to remedy the evils referred to.

### THE DRUG MARKET

Business in drugs and chemicals is now assuming a more normal appearance, and although the demand cannot be termed heavy, there is a fairly good inquiry which should lead to orders in due course. Ipecacuanha is again dearer by 6d. per lb., which makes an advance of about 1s. 3d. per lb. in two weeks. Buchu leaves are also dearer again. Jalap, on the other hand, is slightly cheaper. The reports so far received from the Norwegian cod fisheries are satisfactory, and, provided the weather is favourable, a good catch seems to be promised. The livers are reported to be of fair quality. It is, of course, too early to form an idea as to what the results of the fishing will be, but so far as can be judged at present the indications are that there should be a good catch and a good field of oil, in which case the price of cod liver oil should continue at a normal figure. So much, however, depends on the weather in the fishing grounds that it is not wise to form an opinion on the data at present available. As anticipated in last week's report the price of cocaine is slightly lower. So much uncertainty exists as regards the spirit duties that it would seem unwise to purchase more spirit and preparations containing spirit than is necessary to satisfy the requirements of a few weeks. The increased duty provided for in the Budget will most likely be sanctioned when the new Parliament meets, but the question arises whether some remission of the duty will not be proposed in the Budget for the financial year beginning on April 1 next.

